# Lung Cancer Awareness Among Adults in Riyadh, Saudi Arabia: A Cross‐Sectional Study Using the Lung Cancer Awareness Measure (CAM)

**DOI:** 10.1155/nrp/1059331

**Published:** 2026-07-08

**Authors:** Samah Saad Salem, Raghib Abu-Saris, Moudi Albargawi, Shaykhah Albashir, Rana Abdulwahab Alqarni, Ruyuf Abdullah Altuwayjiri, Renad Naif Alanazi, Shaimaa Mohamed Elhadary, Mona Alanazi

**Affiliations:** ^1^ Medical-Surgical Nursing Department Faculty of Nursing, Cairo University, Cairo, Egypt, cu.edu.eg; ^2^ College of Nursing, King Saud Bin Abdulaziz University for Health Sciences, Riyadh, Saudi Arabia, ksau-hs.edu.sa; ^3^ King Abdullah International Medical Research Center, Riyadh, Saudi Arabia, kaimrc.med.sa; ^4^ Ministry of the National Guard Health Affairs, Riyadh, Saudi Arabia; ^5^ Epidemiology and Biostatistics Department College of Public Health and Health Informatics, King Saud Bin Abdulaziz University for Health Sciences, Riyadh, Saudi Arabia, ksau-hs.edu.sa

**Keywords:** awareness, cross-sectional study, lung cancer, nursing, risk factors, Saudi Arabia

## Abstract

**Background:**

Lung cancer remains a leading cause of cancer‐related mortality worldwide, including in Saudi Arabia. Public awareness of lung cancer symptoms and risk factors is essential for early detection and improved outcomes.

**Objective:**

To assess awareness of lung cancer symptoms, risk factors, and confidence in symptom recognition among Saudi adults in Riyadh using the Lung Cancer Awareness Measure (CAM).

**Methods:**

A cross‐sectional study was conducted among 400 Saudi adults in Riyadh between August and September 2024 using an Arabic‐translated and validated Lung CAM questionnaire. Descriptive statistics, chi‐square tests, and multivariable logistic regression were used for analysis.

**Results:**

High awareness of lung cancer symptoms was observed in 36.3% of participants, and 25.5% demonstrated high awareness of risk factors. Smoking (79.3%) and exposure to secondhand smoke (74.0%) were the most recognized risk factors. Fair confidence of noticing a cancer symptom was reported by 42.8% of the participants, while only 5% reported high confidence. Higher knowledge and awareness of signs and symptoms were significantly associated with having higher knowledge of risk factors, current occupation, and having a close friend with cancer.

**Conclusion:**

Although less than half of the study’s participants showed high awareness of lung cancer symptoms or risk factors, smoking and secondhand smoke exposure were well‐identified by them as key risk factors. Confidence in symptom recognition was remarkably low, with only 5% reporting high confidence. Higher knowledge of signs and symptoms was significantly associated with increased awareness of risk factors, current occupation, and having a close friend with cancer. These findings highlight the need for targeted health education campaigns, mainly for less‐identified lung cancer risk factors and symptoms, to improve early detection and reduce morbidity and mortality, wherein medical‐surgical nurses play a crucial role in bridging this knowledge gap through clinical patient education and symptom‐awareness promotion.

## 1. Introduction

Cancer is the second leading cause of death globally and represents a major and growing public health concern. According to the World Health Organization (WHO), worldwide cancer mortality exceeded 9.6 million deaths in 2018, driven by population aging, lifestyle changes, environmental exposures, and increased life expectancy [1]. In the Arab world, cancer mortality remains a significant challenge. A recent assessment reported that the mortality‐to‐incidence ratio (MIR) among women in Arab countries was comparable to 2018 levels but stayed higher than global averages, highlighting the need for targeted prevention and early detection strategies in the region [2].

Cancer comprises a heterogeneous group of diseases characterized by uncontrolled cellular growth, resulting from complex interactions between genetic susceptibility, environmental exposures, and behavioral factors [3]. Although cancers vary in etiology, clinical presentation, and prognosis, common types, such as breast, lung, colorectal, prostate, and skin cancers, account for a substantial percentage of global morbidity and mortality [4]. Reducing the burden of cancer relies largely on effective prevention strategies. Primary prevention focuses on minimizing exposure to modifiable risk factors, including tobacco use and environmental pollutants, while promoting healthy behaviors and vaccination programs. Secondary prevention emphasizes early detection through screening and symptom recognition, which can significantly improve management outcomes and survival rates [5].

Among all types of malignancies, lung cancer remains one of the most prevalent and deadliest cancers worldwide, primarily resulting from extended exposure to carcinogens, most notably tobacco smoke. Active smoking is the leading risk factor, while exposure to secondhand smoke also contributes substantially to disease development [5]. Despite advances in treatment, lung cancer is frequently diagnosed at advanced stages, underscoring the importance of prevention, early symptom recognition, and timely healthcare seeking [6].

Globally, lung cancer incidence is increasing, particularly in low‐ and middle‐income countries, largely due to rising tobacco use and industrialization, with the average age at diagnosis being approximately 70 years [7]. Common warning signs of lung cancer include persistent cough, hemoptysis, chest pain, and shortness of breath; however, these symptoms are often overlooked or misattributed, leading to delayed diagnosis [6].

Lung cancer screening is crucial in reducing lung cancer mortality among high‐risk populations. The American Cancer Society estimated 135,720 lung cancer deaths in 2020, prompting updates to screening guidelines, including lowering the eligibility age and smoking exposure thresholds [8]. Nevertheless, access to screening and public awareness of its benefits remain limited in many countries, particularly in resource‐limited settings [9]. Managing lung cancer continues to pose a significant challenge, reinforcing the importance of prevention and early detection strategies [10].

In the Middle East and North Africa (MENA) regions, lung cancer represents an expanding health burden, with lung cancer accounting for 23.9% of cancers among men in countries such as Algeria, the Gulf Cooperation Council (GCC) countries, Jordan, Morocco, Lebanon, and Tunisia [11]. For lung cancer detection, particularly among high‐risk individuals, low‐dose computed tomography (CT) scans demonstrate high sensitivity and specificity [12]. The increasing prevalence of smoking among younger populations suggests a continued rise in lung cancer incidence in the coming decades. Globally, approximately two million lung cancer cases and deaths were reported in 2020, with estimates projecting up to 3.8 million new cases annually by 2050 if current trends persist [13]. Expanding screening programs, refining risk assessment models, and enhancing public awareness are crucial to improving outcomes [14, 15].

In Saudi Arabia, lung cancer remains a significant and escalating public health challenge. It is the fifth most diagnosed cancer and the third leading cause of cancer‐related mortality nationwide [6]. Among Saudi men, lung cancer ranks fifth in incidence, with rates ranging from 1.2 to 12.3 per 100,000, while among women it ranks ninth, with rates between 0.2 and 3.1 per 100,000. Evidence indicates that men are approximately three times more likely to develop lung cancer than women, mainly due to higher smoking prevalence. Age is also a critical factor, with nearly 25% of cases occurring in individuals older than 75 years [16]. Projections indicate an 87% increase in lung cancer cases between 2013 and 2030, posing a substantial challenge to the healthcare system [17].

Despite the rising burden of lung cancer, awareness of its risk factors, symptoms, and early warning signs remains limited among the Saudi population. Previous studies recommend that limited knowledge of cancer prevention and symptom recognition contributes to delayed diagnosis and worse outcomes [18]. Moreover, regional differences in incidence emphasize the need for localized awareness initiatives, with the Eastern region reporting the highest age‐related incidence rates among both genders, followed by Riyadh and other regions [19]. Nurses play a central role in health education, early detection, and the assessment of public awareness, all of which are particularly relevant to nursing practice.

Although lung cancer is a leading cause of cancer morbidity and mortality in Saudi Arabia, evidence on public awareness—especially among adults in Riyadh—remains scarce. Understanding the population’s awareness of lung cancer symptoms and risk factors, as well as their confidence in recognizing symptoms, is crucial for designing effective nursing educational interventions and public health strategies [6]. Therefore, this study aims to assess lung cancer awareness regarding symptoms, risk factors, and confidence in symptom recognition among Saudi adults in Riyadh, as well as to examine associated demographic factors.

## 2. Aim of the Study

To assess lung cancer awareness regarding symptoms, risk factors, and confidence in symptom recognition among Saudi adults in Riyadh, and to examine associated demographic factors.

## 3. Research Questions

• What is the level of lung cancer awareness as regards warning signs and symptoms among Saudi adults in Riyadh?
• What is the level of lung cancer awareness as regards risk factors among Saudi adults in Riyadh?
• What is the level of lung cancer awareness as regards confidence in detecting symptoms among Saudi adults in Riyadh?
• What factors are associated with lung cancer awareness among Saudi adults in Riyadh?

## 4. Materials and Methods

### 4.1. Study Design and Setting

A cross‐sectional study design was utilized to collect data from the representative sample. This design is used to examine the relationships between multiple variables [20]. Data collection took place from August to September 2024. This study was conducted in Riyadh, the capital of Saudi Arabia, with a population of approximately 8 million. The researchers used an online survey to assess social and health‐related factors to update targeted activities aimed at improving lung cancer awareness. Riyadh’s diverse demographic profile provided an ideal setting to explore the city’s unique challenges and resources related to lung cancer awareness.

### 4.2. Participants and Sampling

**FIGURE 1:**
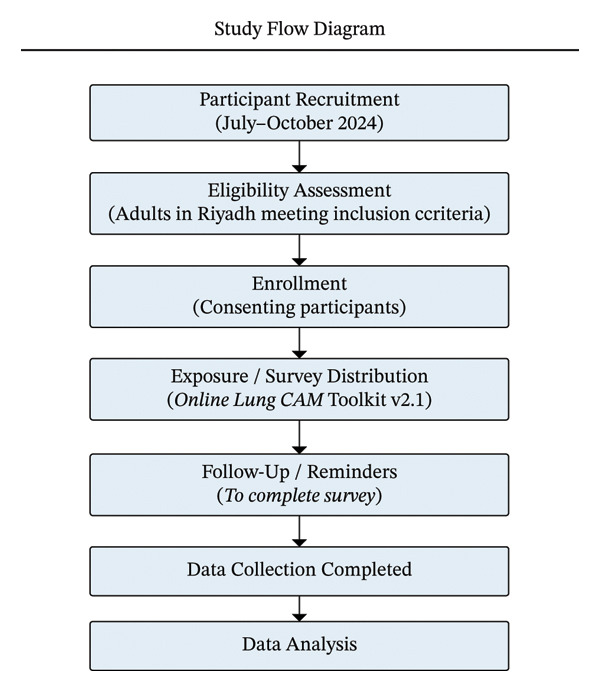
Study flow diagram.

To achieve the aim of the current study, 400 participants were recruited. Eligible participants were Saudi adults aged 18–64 years living in Riyadh, representing a range of educational levels and occupational sectors. The sample size was calculated using statistical software, considering an alpha level of 0.05, a statistical power of 0.80, a 5% margin of error, and a 95% confidence level. The calculation indicated a minimum sample size of 385, but 400 participants were recruited to ensure a more representative sample. A consecutive sampling technique was used. The survey was created using Google Forms and distributed via WhatsApp. While this approach facilitated rapid data collection, it may have introduced selection bias, as participation was limited to individuals who were digitally active and socially connected. Consequently, the findings may not fully represent the general adult population in Riyadh. Measures were taken to ensure that each participant could submit only one response. The inclusion criteria were clearly displayed before participation (see Figure 1).

### 4.3. Data Collection Tool

To collect the required data for this study, the Lung Cancer Awareness Measure (Lung CAM) Toolkit Version 2.1, a web‐based survey, was utilized. Developed by University College London and Cancer Research UK, the Lung CAM survey included two parts: the first part gathered demographic data (age, gender, ethnicity, language, marital status, and education level). The second part comprised seven sections with 28 questions on warning signs, risk factors, and confidence in detecting symptoms [21].

The survey included closed (yes/no) and open‐ended questions to comprehensively assess lung cancer awareness, with open‐ended questions providing deeper insights into participants’ knowledge and experiences. The toolkit, validated for internal consistency and content validity, effectively evaluated awareness and risk factors [22, 23]. The Lung CAM demonstrated good internal (Cronbach’s *α* = 0.88) and test–retest reliability (*r* = 0.81, *p* < 0.001). Validity was supported by lung cancer experts scoring higher than equally educated controls (*t* (106) = 8.7, *p* < 0.001) and volunteers randomized to read lung cancer information scoring higher than those reading a control leaflet (*t* (81) = 3.66, *p* < 0.001) [24].

The original survey was developed in English, and for this research, it was translated into Arabic using modern translation software and the forward‐backward method as recommended by WHO [25] to ensure cultural and linguistic appropriateness. The Lung CAM Toolkit is freely available for research purposes without a needed permission, and survey translation is also permitted without restriction.

### 4.4. Validity and Reliability

A pilot study including 40 participants was conducted to evaluate the readability and suitability of the Arabic version. Bilingual experts reviewed the translation and performed validity and reliability assessments. The mean reliability of all items revealed strong internal consistency, with a value of 0.96. The agreement level ranged from very good to excellent, with an average intraclass correlation coefficient (ICC) of 0.867 and a 95% confidence interval (CI) of (0.8508, 0.8803). We verify that all translated versions have been validated to ensure their reliability and validity.

### 4.5. Data Analysis

Data were analyzed using SPSS Version 22. Categorical variables were described using frequencies and percentages, while numerical variables were summarized using means and standard deviations or medians and interquartile ranges (IQR). Lung cancer awareness levels were estimated with a 95% CI. Chi‐square tests, Fisher’s exact test, and independent sample *t*‐tests were performed to examine relationships between research variables and demographic characteristics. To assess the strength of associations, a multivariable regression analysis was employed. A significant level of 0.05 was applied for all statistical analyses.

### 4.6. Ethical Considerations

Approval was obtained from the Research Unit of the College of Nursing (CON) at King Saud bin Abdulaziz University for Health Sciences (KSAU‐HS) and the King Abdullah International Medical Research Center (KAIMRC) before commencing the study (IRB Approval No.: 0000012624). Eligible volunteers were informed about the study’s objectives, assured that participation was voluntary, and given the option to withdraw at any time without any consequences. Participant privacy was prioritized by ensuring confidentiality, anonymity, and the nondisclosure of personal information. The principal investigator ensured the secure storage of all data.

## 5. Results

### 5.1. Sociodemographic Data

Data were collected from 400 participants aged 18 to 64 and analyzed using SPSS Version 25.0. Of the 400 participants, 42.8% reported fair confidence, while only 5% were very confident in noticing a symptom of lung cancer.

Most participants were female (88.5%), single (78.3%), and nonsmokers (93.3%), with a mean age of 24.87 years. Most participants had either a college degree or higher degrees (64.8%), were still studying (61.8%), or had become aware of cancer through personal experience, family, or friends (71.5%). For further details, see Table 1.

**TABLE 1:** Sample demographics (*n* = 400).

Variable	Categories	Frequency (percentage)	Mean (SD)	Median (IQR)
Age			24.87 (9.03)	21.00 (6.75)

Gender	Male	46 (11.5)		
	Female	354 (88.5)		

Marital status	Single	313 (78.3)		
	Married	76 (19.0)		
	Widow	3 (8.0)		
	Divorced	8 (2.0)		

Education level	Less than primary	1 (0.3)		
	Secondary	140 (35.0)		
	College and higher	259 (64.8)		

Occupation	Employed	64 (16.0)		
	Unemployed	82 (20.5)		
	Retired	7 (1.8)		
	Still studying	247 (61.8)		

Smoking	Yes	23 (5.8)		
	No	373 (93.3)		
	Don’t know	4 (1.0)		

Cancer acquaintance	No	114 (28.5)		
	Yes	286 (71.5)		

Confidence in noticing a lung cancer symptom	Not all confident	63 (15.8)		
	Not very confident	146 (36.5)		
	Fairly confident	171 (42.8)		
	Very confident	20 (5.0)		

**FIGURE 2:**
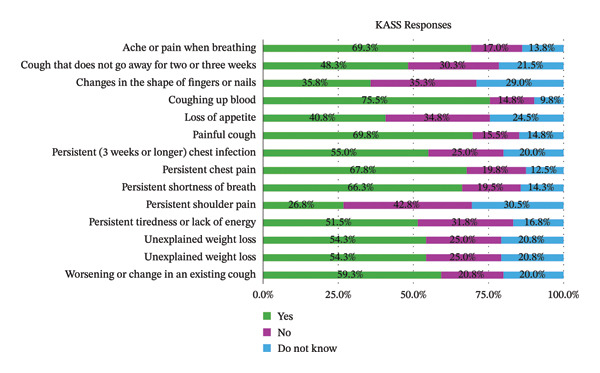
Distribution of KASS responses (*n* = 400).

Awareness of warning signs and symptoms was highest for coughing up blood, painful cough, and pain when breathing (75.5%, 69.8%, and 69.3%, respectively), while the lowest awareness was for loss of appetite, changes in the shape of fingers or nails, and persistent shoulder pain (40.8%, 35.8%, and 26.8%, respectively) (Figure 2).

**FIGURE 3:**
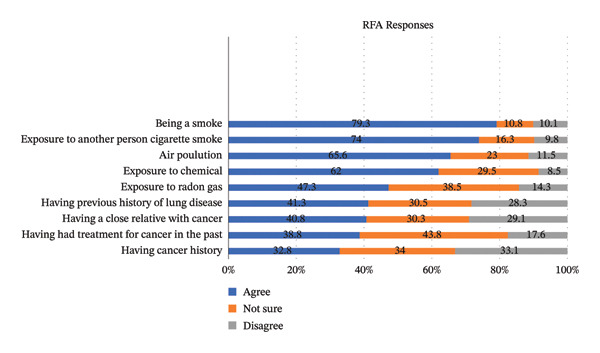
Distribution of RFA responses (*n* = 400).

Regarding awareness of risk factors, Figure 3 illustrates that the highest responses were for being a smoker, exposure to another person’s cigarette smoke, and air pollution (79.3%, 74%, and 65.6%, respectively). In contrast, the lowest responses were for having a close relative with cancer, having undergone treatment for cancer in the past, and having a personal history of cancer (40.8%, 38.8%, and 32.8%, respectively).

Like Estifanos et al. [26], the 14 lung cancer symptom awareness indicator variables were dichotomized into either “yes” or “no (including don’t know).” Similarly, the nine lung‐cancer potential risk‐factor indicators were dichotomized into “yes” for agree or disagree and “no” otherwise. In addition, high awareness means the percentage of “yes” is ≥ 70% [5]; otherwise, it is low awareness.

A little above one‐third of the participants (36.3%; 95%CI: 31.5%–41.0%) demonstrated a high level of symptoms knowledge and awareness (KASS). On the other hand, about one‐quarter of the participants (25.5%; 95%CI: 21.2%–29.8%) depicted risk‐factor awareness (RFA). For more details, see Table 2.

**TABLE 2:** Point and interval estimations of knowledge and awareness of signs and symptoms (KASS) and risk factors awareness (RFA).

Variable	Category	Estimate %	95% confidence interval
Awareness of lung cancer signs and symptoms	High	36.3	(31.5%, 41.0%)
	Low	63.7	(59.0%, 68.5%)

Awareness of lung cancer risk factors	High	25.5	(21.2%, 29.8%)
	Low	74.5	(70.2%, 78.8%)

Using the *χ* ^2^‐test for independence or Fisher’s exact test, as appropriate, we identified factors that were significantly (*p* value < 0.05) associated with KASS or RFA (see Table 3 below). It turns out that none of the demographic factors were significantly associated with RFA, indicating statistically comparable distribution of RFA across the different categories, while marital status, current occupation, personal history of cancer, family history of cancer, and close friends with cancer were significantly associated with KASS.

**TABLE 3:** Factors significantly associated with KASS or RFA.

Variable	Category	KASS			RFA		
		Low	High	*p* value	Low	High	*p* value
Age (mean ± SD)	—	25.61 ± 9.88	23.56 ± 7.14	**0.017** ^∗∗^	24.94 ± 9.44	24.65 ± 7.75	0.757^∗∗^

Marital status	Single	189	124	**0.010** ^∗^	232	81	0.672^∗^
	Married	55	21		58	18	
	Widow	3	0		3	0	
	Divorced	8	0		5	3	

Current occupation	Employed	39	25	0.037**^∗^**	46	18	0.869
	Unemployed	62	20		61	21	
	Retired	6	1		6	1	
	Still studying	148	99		185	62	

Close friend has cancer	Yes	21	23	**0.012**	30	14	0.190
	No	68	254		244	78	
	Don’t know	11	22		23	10	

*Note:* *p* values in bold indicate statistical significance (*p* < 0.05).

^∗^Using Fisher’s exact test.

^∗∗^Using an independent samples *t*‐test.

On the other hand, KASS and RFA were significantly associated (*p* value < 0.001); see Table 4.

**TABLE 4:** Association between KASS and RFA (McNemar’s test).

		KASS		
		Low	High	*p* value
RFA	Low	221	77	**< 0.001**
	High	34	68	

*Note:* Bold indicates statistical significance at *p* < 0.05. McNemar’s test showed a statistically significant association between KASS and RFA (*p* < 0.001).

To evaluate the strength of the association between factors significantly associated with KASS, we employed a multivariable binary logistic regression. The results for KASS are presented in Table 5. It was found that RFA, current occupation, and having a close friend with cancer significantly influenced the likelihood of high KASS. The logistic regression model demonstrated a good fit, as indicated by the Hosmer–Lemeshow test (*p* value = 0.572 > 0.05). The model explained 31.2% of the variance in KASS (Nagelkerke R^2^). Specifically, awareness of cancer risk factors increased the odds of high KASS by more than sixfold. Additionally, not having or not knowing a close friend with cancer decreased the odds of high KASS by more than 55%. Likewise, the odds of high KASS among the unemployed are approximately 55% less than those of the employed (Table 5).

**TABLE 5:** Multivariable binary logistic regression for KASS.

Variable	Categories	Coef.	*p* value	aOR	95% C.I. OR	
					Lower	Upper
Age	—	0.004	0.771	1.004	0.976	1.033

RFA (Ref. = No)	Yes	1.846	**< 0.001**	6.332	3.803	10.543

Marital status (Ref. = Single)	Married/Widow/Divorced	−0.830	0.061	0.436	0.183	1.040

Current occupation (Ref. = Employed)	Unemployed	−0.801	**0.037**	0.449	0.212	0.952
	Retired	−1.390	0.281	0.249	0.020	3.112
	Still studying	−0.179	0.565	0.836	0.454	1.539

Close friend has cancer (Ref. = Yes)	No	−0.819	**0.015**	0.441	0.228	0.854
	Do not know	−1.214	**0.024**	0.297	0.103	0.853

*Note:* *p* values in bold indicate statistical significance (*p* < 0.05). Ref., reference category; Coef., regression coefficient.

Abbreviations: aOR, adjusted odds ratio; CI, confidence interval.

Similarly, KASS was the only factor found to significantly affect the likelihood of RFA. The logistic regression model demonstrated a good fit and explained 18.4% of the variance in RFA (Nagelkerke R^2^). Specifically, a higher KASS increased the odds of RFA by more than fivefold (Table 6).

**TABLE 6:** Multivariable binary logistic regression for RFA.

Variable	Categories	Coef.	*p* value	OR	95% C.I. OR	
					Lower	Upper
KASS (Ref. = No)	Yes	1.748	**< 0.001**	5.740	3.529	9.338

*Note:* *p* values in bold indicate statistical significance (*p* < 0.05). Ref., reference category.

Abbreviations: CI, confidence interval; OR, odds ratio.

## 6. Discussion

The current study assessed lung cancer awareness among Saudi adults in Riyadh and found that fewer than half demonstrated high awareness of symptoms (36.3%) or risk factors (25.5%), with only 5% reporting high confidence in identifying those symptoms. Awareness was further affected by occupation, having a close friend with cancer, and overall risk factor knowledge. These findings emphasize a noteworthy gap in lung cancer awareness and highlight the need for targeted health initiatives to promote early detection and improve outcomes.

Regarding symptom awareness, fewer than half of the participants demonstrated high awareness of lung cancer symptoms, which matched the results of Alrabeeah et al. [5], who reported that overall awareness of lung cancer signs and symptoms was 53%. More severe symptoms, such as coughing up blood, a painful cough, and pain when breathing, were commonly identified, while less typical signs, such as loss of appetite, changes in the shape of fingers or nails, and persistent shoulder pain, remained less recognized in the current study. This knowledge gap is clinically significant, as delayed recognition of atypical symptoms may lead to late‐stage diagnosis and reduced treatment outcomes, as individuals may not seek medical help until more severe symptoms exist. Matched with these results, Estifanos et al. [26]. reported that only one‐fourth of participants demonstrated good awareness of lung cancer symptoms, with respiratory symptoms being the most identified, and awareness varied across sociodemographic differences, strengthening the need for targeted, class‐specific educational interventions with a particular focus on nonpulmonary symptoms. In the same line, consistent painful cough and coughing up blood are the best‐known symptoms. Conversely, persistent shoulder pain (44%) and clubbing fingers (47%) were the least known lung cancer symptoms [5].

Regarding confidence in identifying symptoms, only 5% of the study participants reported high confidence, while less than half of them reported fair confidence. Similar results reported by Alrabeeah et al. [5] showed 60% of the respondents showed low confidence in identifying the signs and symptoms of lung cancer. Participants with higher awareness of risk factors demonstrated greater confidence in symptom recognition. Supports the idea that Saab et al. [27] emphasized that educational interventions play an important role in enhancing confidence in symptom detection. The current study results also showed that having a close friend with cancer was significantly associated with increased awareness of lung cancer symptoms and risk factors, signifying that personal connections play an important role in health knowledge gaining. Opposite to this, Abdel‐Razeq et al. reported that no significant difference in modifiable risk factors was found between participants with a friend who has cancer and those who do not (*p* = 0.193) [28]. In agreement with the same result, all responses for recognizing risk factors saw a significant improvement in how participants responded after, except for smoking (*p* = 0.453), passive smoking (*p* = 0.180), and having a close relative with cancer (*p* = 0.344) [29]. Conversely, participants who knew someone with cancer were more likely to recognize ‘exposure to chemicals’, ‘exposure to radiation’, ‘air pollution’, ‘having a previous history of cancer’, and ‘having had treatment for any cancer in the past’ as LC risk factors as identified by Elshami et al. [30]. To maximize the impact, public health efforts and nursing awareness interventions in Riyadh should integrate personal histories with evidence‐based education to bridge the gaps in knowledge, encourage early detection, and improve outcomes.

The current study showed that only one‐quarter of participants demonstrated high RFA, indicating a considerable lung cancer risk factor knowledge gap among Saudi adults in Riyadh. Smoking and exposure to secondhand smoke were the most widely recognized risk factors among the current study participants, aligned with Alrabeeah et al. [5], who reported the same higher public awareness of smoking‐related risks. These results were verified by findings where smoking cigarettes (93.8%) and smoking shisha (91.1%) were the most identified risk factors, strengthening the idea that smoking‐related risks are consistently better recognized across different populations and countries [30]. However, awareness of nonsmoking‐related risk factors, such as exposure to air pollution, chemicals, and radon, remained significantly lower in the present study. Williams et al. [29] also emphasized that many nonsmokers develop lung cancer due to environmental exposures such as radon and domestic fuel smoke, emphasizing the clinical significance of this knowledge gap.

The study demographics revealed that most participants were female and nonsmokers, consistent with global trends showing lower smoking rates among women compared to men. This pattern may be explained by a combination of social, cultural, and economic influences. In conservative societies such as Saudi Arabia, smoking among women may be socially discouraged, which can reduce its prevalence. Moreover, women may be more cautious about smoking due to higher awareness of its health consequences, mainly those related to pregnancy and reproductive health. Economic factors may also limit smoking behaviors among women in certain contexts. The predominance of female participants in this study should be considered when interpreting the findings, as it may have influenced the observed levels of awareness and limited the applicability of the results to male populations.

Finally, multivariable binary logistic regression of the current study revealed that RFA, current occupation, and having a close friend with cancer were significant independent predictors of KASS, while age and marital status were not significant predictors. This highlights the strong bidirectional relationship between symptom and risk factors awareness, suggesting that these are commonly supporting concepts and that interventions directing one domain are most likely to lead to increases in the other. This finding matched with previous research study results reported that awareness of lung cancer risk factors and symptoms is significantly shaped by sociodemographic variables, including age, education, and employment status, highlighting the multidimensional nature of cancer health awareness in the general population [5]. For the occupational status, unemployed participants had significantly lower odds of high symptom awareness compared to employed participants, indicating that the workplace may serve as an important channel for health awareness and education delivery. These findings are consistent with prior research evidence suggesting that employment, aligned with higher educational attainment and income, is associated with a positive impact on cancer awareness, most likely due to greater exposure to health‐related knowledge and professional environments that enhance the health‐promoting behaviors [30]. Regarding the social influence of KASS, participants who did not have a close friend with cancer demonstrated significantly lower symptom awareness. This validates findings indicating that knowing someone having cancer, either a close friend or relative, positively enhances the awareness levels, possibly by personal exposure to the cancer manifestations throughout the cancer journey, including recognition of its early warning signs [30]. In conclusion and taking all points together, these findings suggest that modifiable factors, especially risk factor knowledge and personal cancer exposure, have a stronger influence on symptom awareness than demographic characteristics only, highlighting the need for increased awareness campaigns that reach beyond age‐defined groups to address broader awareness gaps in the larger population.

## 7. Conclusion

The current study results reveal a large gap in lung cancer awareness among Saudi adults in Riyadh, with less than half of the study’s participants demonstrating high KASS and only one‐quarter of them demonstrating high RFA. While common and severe symptoms such as hemoptysis, persistent cough, and shortness of breath were moderately well identified, less typical symptoms, such as loss of appetite, changes in finger or nail shape, and shoulder pain, remained poorly recognized. Confidence in recognizing the symptoms was particularly low, with only a small minority of participants reporting high confidence, highlighting that the only knowledge is inadequate without structured awareness campaigns and interventions. Regarding RFA, smoking and exposure to secondhand smoke were the most acknowledged, whereas awareness of nonsmoking‐related factors, such as exposure to air pollution, asbestos, and radon, and the presence of family history, is still limited. Age was frequently not recognized as a lung cancer risk factor, which may contribute to delayed diagnosis among the elderly. Multivariable analysis identified RFA, current occupation, and having a close friend with cancer as significant independent predictors of KASS, while age and marital status were not significant, suggesting that modifiable knowledge‐based factors have a higher impact than demographic characteristics only. These emphasize the need for comprehensive, culturally responsive health awareness campaigns and nursing education that address both common and lesser‐known lung cancer symptoms and risk factors, target employed and unemployed populations through appropriate health awareness channels, and influence personal cancer narratives with structured, evidence‐based cancer education to promote lung cancer early detection and improve the outcomes across all demographic groups in Riyadh, Saudi Arabia.

## 8. Implications for Practice

The findings of this study highlight the critical role of nurses in enhancing lung cancer awareness and promoting early detection. To translate these findings into practice, several actionable nurse‐led interventions can be implemented. At the clinical level, nurses can integrate brief lung cancer risk assessments into routine triage protocols, particularly in primary care and outpatient settings, to identify high‐risk individuals such as smokers or those exposed to environmental risk factors. In addition, nurses can deliver structured health education sessions in waiting areas, focusing on early warning signs, less‐recognized symptoms, and the importance of timely medical consultation. Nurses also play a key role in smoking cessation counseling, providing evidence‐based interventions and referrals to cessation programs. Incorporating lung cancer education into routine patient interactions, such as during chronic disease follow‐ups, can further reinforce awareness.

At the community level, nurses can lead outreach initiatives and awareness campaigns through schools, workplaces, and community centers, with a focus on both smoking‐related and nonsmoking‐related risk factors. Collaboration with public health organizations can support the development of targeted educational materials tailored to specific demographic groups, including males and underserved populations. Furthermore, nurses can contribute to early detection efforts by promoting awareness of screening eligibility criteria and facilitating referrals for appropriate diagnostic evaluation when indicated. Implementing these nurse‐led strategies can enhance public awareness, improve early symptom recognition, and ultimately contribute to earlier diagnosis and better health outcomes.

## 9. Recommendation

• **Developing and implementing targeted public health campaigns:** These campaigns should focus on educating the public about lung cancer risk factors, early detection signs and symptoms, and the critical importance of early diagnosis and treatment. Tailoring the content to the specific needs and concerns of the population will enhance the effectiveness of the campaign.
• **Utilizing diverse communication channels:** Information should be disseminated through multiple platforms, including social media, community outreach programs, and educational initiatives led by specialized medical‐surgical nurses. This multichannel approach ensures the widest possible reach and engages a large audience effectively.
• **Addressing health disparities:** Public health efforts should prioritize reaching underserved populations and addressing disparities in lung cancer awareness and outcomes, both within Saudi Arabia and in neighboring regions. Targeting vulnerable groups will help reduce the gap in awareness and health outcomes.
• **Conducting further research:** Continued research is essential to evaluate the effectiveness of various awareness campaigns. Exploring innovative strategies to enhance lung cancer knowledge and facilitate early detection will further strengthen public health efforts.

## 10. Limitations

This study has several limitations. First, the use of a convenience (purposive) sampling method via WhatsApp may have introduced selection bias, as participants were more likely to be younger, educated, and digitally active, thereby limiting the generalizability of the findings to the broader adult population in Riyadh. Second, the sample was predominantly female (88.5%), representing a substantial gender imbalance. This may have introduced gender‐related bias, as women are generally more likely to participate in health‐related surveys and may exhibit higher levels of health awareness. Consequently, this imbalance may have led to an overestimation of lung cancer awareness, particularly when generalizing the findings to male populations. Third, the cross‐sectional design restricts the ability to establish causal relationships between awareness levels and associated factors. Despite these limitations, the study provides valuable insights into lung cancer awareness among Saudi adults and highlights important gaps in symptom recognition and understanding of risk factors. Future research using probability‐based sampling methods and longitudinal designs, along with more refined measurement approaches (e.g., continuous awareness scoring), is recommended to enhance the robustness and generalizability of the findings.

## Funding

No funding was received for this manuscript.

## Conflicts of Interest

The authors declare no conflicts of interest.

## Data Availability

The data that support the findings of this study are available from the corresponding author upon reasonable request.
